# Novel molecular data for monogenean parasites of sparid fishes in the Mediterranean and a molecular phylogeny of the Microcotylidae Taschenberg, 1879

**DOI:** 10.1016/j.crpvbd.2021.100069

**Published:** 2021-12-24

**Authors:** Lamia Lablack, Mohammed Rima, Simona Georgieva, Douniazed Marzoug, Aneta Kostadinova

**Affiliations:** a Laboratoire Réseau de Surveillance Environnementale, Faculté des Sciences de la Nature et de la Vie, Université Oran 1 Ahmed Ben Bella, Département de Biologie, 31000 Oran, Algeria; b Département en Eau Environnement et Développement Durable, Faculté des Sciences de la Nature et de la Vie, Université Hassiba BenBouali de Chlef, B.P 78C, Ouled Fares 02180, Chlef, Algeria; c Institute of Biodiversity and Ecosystem Research, Bulgarian Academy of Sciences, 2 Gagarin Street, 1113 Sofia, Bulgaria; d Institut Cavanilles de Biodiversitat i Biologia Evolutiva, Parc Científic, Universitat de València, PO Box 22085, Valencia 46071, Spain

**Keywords:** Monogenea, Microcotylidae, Capsalidae, Diclidophoridae, Molecular phylogeny, 28S rDNA, *cox*1

## Abstract

During a study of the monogeneans of four sparid fishes (*Diplodus vulgaris*, *Pagellus bogaraveo*, *Pagrus pagrus* and *Sparus aurata*) from the Western Mediterranean off Algeria, a large collection of parasites was characterised molecularly (28S rRNA and *cox*1 genes). A total of 46 partial sequences (23 for each gene) were generated from 38 isolates of monogeneans which included four species (*Atrispinum acarne*, *Microcotyle erythrini* (*sensu stricto*), *Sparicotyle chrysophrii* and Prostatomicrocotylinae gen. sp.) of the family Microcotylidae, two putative species of the family Capsalidae (*Encotyllabe* spp.), and one species (*Choricotyle chrysophryi*) of the family Diclidophoridae. Our study provides (i) the first molecular data for the Capsalidae in the Mediterranean; (ii) the first record of a member of the Prostatomicrocotylinae in the Mediterranean and in a sparid fish (*D. vulgaris*); (iii) the first *cox*1 sequences for *A. acarne*; (iv) the second record of *M. erythrini* (*s.s.*) from *P. pagrus*; and (v) the second confirmed by molecular data record of *S. chrysophrii* in wild populations of *S. aurata.* The first phylogenetic hypotheses for the family Microcotylidae developed here, revealed the monophyly of the subfamily Prostatomicrocotylinae and the genus *Microcotyle* but the relationships among the subfamilies were still largely unresolved with the best represented subfamily Microcotylinae being polyphyletic. Our results highlight the importance of molecular methods in the assessment of monogenean diversity and the need for a thorough taxon-sampling approach to increase the accuracy of phylogenetic reconstruction of the relationships of the large and taxonomically complex polyopisthocotylean family Microcotylidae.

## Highlights

• Novel sequence data for monogeneans of sparid fishes in the Mediterranean.
• First molecular phylogenies of the Microcotylidae based on 28S rRNA and *cox*1 genes.
• The relationships among the subfamilies of the Microcotylidae are still largely unresolved.
• First record of a member of the Prostatomicrocotylinae in the Mediterranean and in a sparid fish.
• Two putative species of *Encotyllabe* (family Capsalidae).

## Graphical abstract

**Figure:**
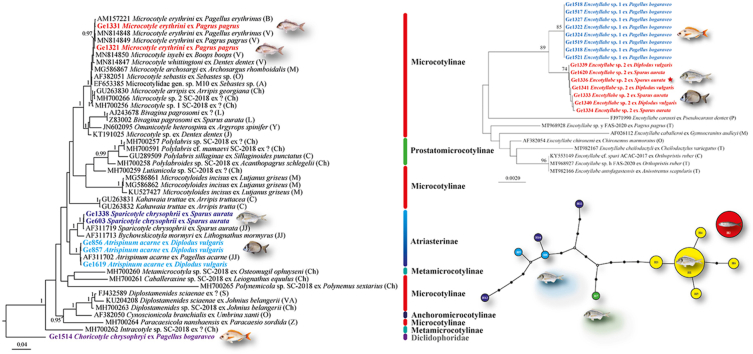

## 1 Introduction

The Mediterranean is one of the first marine areas where parasite diversity has been explored and is a significant area of species occurrence and endemism of fish hosts and their parasites. Parasite diversity in Mediterranean fishes is generally well surveyed, especially for the most diverse host family (31 spp.), the Sparidae (see Pérez-del-Olmo et al., 2016, for a recent review of digenean diversity). In the Mediterranean, sparid fishes are important commercial and recreational angling species and a promising source for the diversification of marine aquaculture (Basurco et al., 2011). However, culturing at high density inevitably leads to parasite-related problems associated with spill-over from and to wild fish populations aggregating around sea-cage facilities in coastal areas (see, e.g. Bouwmeester et al., 2020 for a recent review).

Monogeneans are a large group of pathogens with a direct life-cycle causing significant problems in farmed fish and thus may affect the success of the attempts to diversify sparid sea-cage cultures (e.g. Mladineo & Maršić-Lučić, 2007; Sánchez-García et al., 2015). A notable example is the microcotylid *Sparicotyle chrysophrii* (van Beneden & Hesse, 1863) representing a major parasitological problem in sparid cultures in the Mediterranean. There are several reports of lethal epizootics in Mediterranean cultures of *Sparus aurata* L. (see Sánchez-García et al., 2015 and references therein). Therefore, accurate identification of monogeneans in wild sparid populations in areas prospective for aquaculture is important as it provides the baseline data for local pathogen diversity.

However, monogenean identification requires careful morphological studies and taxonomic expertise but similar to other platyhelminth groups, progressively fewer expert morphologists and taxonomists are being trained (Brooks, 2000). Molecular-based methods may provide a standardised framework for diversity assessment in understudied higher taxa and/or areas that is particularly promising for accelerating identification of previously characterised monogenean species where identification based on morphology is complicated (e.g. Bouguerche et al., 2019a, b; Ono et al., 2020; Víllora-Montero et al., 2020). The success of the latter approach depends on the existence of comprehensive sequence databases at least for the most diverse and widely distributed monogenean higher-level taxa.

Regarding sparid fishes worldwide, the polyopisthocotylean family Microcotylidae Taschenberg, 1879 is one such group characterised by a remarkable diversity (more than 160 species in 50 genera of 7 subfamilies; see Mamaev, 1986; WoRMS, 2021) and cosmopolitan distribution. However, although sequences generated for microcotylid species in the Mediterranean have been instrumental in the pioneer phylogenies of the Polyopisthocotylea (see Mollaret et al., 2000; Jovelin & Justine, 2001), the available genetic data for monogeneans of sparid fishes are scarce both globally and for the Mediterranean, and this may hinder diversity assessments for understudied areas and hosts and pathogen identification in aquaculture.

During a study of the monogeneans of sparid fishes from the western Mediterranean off Algeria, a large collection of parasites, which included four species of the family Microcotylidae, two putative species of the family Capsalidae Baird, 1853, and one species of the family Diclidophoridae Cerfontaine, 1895, was characterised molecularly (28S rRNA gene and *cox*1 gene). We show that both markers are effective for identification to the species level within the Microcotylidae and highlight a potential problem for the use of *cox*1 to differentiate *Encotyllabe* spp. Finally, we used the currently available DNA sequence data to explore the phylogenetic relationships within the most speciose polyopisthocotylean family, the Microcotylidae.

## 2 Materials and methods

### 2.1 Sample collection

Four species of sparid fishes [*Pagrus pagrus* (L.) (*n* = 308); *Pagellus bogaraveo* (Brünnich) (*n* = 315); *S. aurata* L. (*n* = 420); and *Diplodus vulgaris* (Geoffroy Saint-Hilaire) (*n* = 390)] collected at four locations along the Algerian coasts of the Western Mediterranean (off Bouzedjar (35°34′24.58″N, 1°10′2.63″W); off Algiers (Bay of Algiers) (36°46′17.60″N, 3°03′57.00″E); off Skikda (36°53′15.94″N, 6°54′0.86″E); and off Annaba (36°57′23.03″N, 7°54′3.88″E)] were examined for parasite infections. Fishes were purchased between 2013 and 2016 from local fishermen during spring and autumn and subsequently examined at the Laboratory of the Environmental Monitoring Unit (LRSE), University of Oran 1, Algeria. Fishes were identified according to Bauchot & Hureau (1986) and examined fresh or at a later stage, after immediate freezing upon collection. Gills and pharyngeal tooth pads were removed, placed in individual Petri dishes, washed in saline solution (9 g NaCl/l) and examined for monogenean parasites under a stereomicroscope. Monogeneans were killed and preserved in molecular biology grade ethanol. Monogenean specimens were subsampled from the total collection and photomicrographs were made for each specimen selected for DNA isolation using an Olympus BX51 microscope (Olympus Corporation, Tokyo, Japan) with the aid of Quick Photo Camera 2.3 image analysis software. A small piece of the body just above the haptor was excised for DNA extraction. The voucher specimens (hologenophores *sensu* Pleijel et al., 2008) were stained with iron acetocarmine (Georgiev et al., 1986), dehydrated in a graded ethanol series (70–100%), cleared in dimethyl phthalate and mounted in Canada balsam. Voucher material is deposited at the Helminthological Collection of the Institute of Biodiversity and Ecosystem Research, Bulgarian Academy of Sciences, Sofia, Bulgaria, under the accession numbers IBER-BAS M0160.1.1-M0160.1.20; IBER-BAS M0160.2.1-M0160.2.19; and IBER-BAS M0160.3.1–3.4.

### 2.2 Sequence data generation

Genomic DNA (gDNA) was isolated using 5% suspension of deionised water and Chelex®, containing 0.1 mg/ml proteinase K; samples were incubated at 56 °C for 3 h, boiled at 90 °C for 8 min, and centrifuged at 14,000× *g* for 10 min. Partial sequences of the nuclear 28S rRNA gene (domains D1-D3) and the mitochondrial cytochrome *c* oxidase subunit 1 gene (*cox*1) were amplified as follows. Partial fragments of the 28S rRNA gene were amplified using primer combinations LSU5 (Littlewood et al., 2000) and 1500R (Tkach et al., 1999) (amplification profile: 5 min at 94 °C, followed by 40 cycles of 94 °C for 30 s, 55 °C for 30 s, 72 °C for 2 min, and a final extension step at 72 °C for 7 min).

Partial fragments of the *cox*1 gene were amplified using the primers JB3 (= COI-ASmit1) and JB4.5 (Bowles et al., 1992) (amplification profile: 5 min at 94 °C, followed by 35 cycles of 94 °C for 1 min, 50 °C for 1 min, 72 °C for 1 min, and a final extension step at 72 °C for 10 min).

PCR amplifications were performed in a total volume of 25 μl using Ready-To-Go-PCR Beads (GE Healthcare, Chicago, USA) following the manufacturer’s instructions. In the case of poor amplification, the PCR reactions were performed with 2× MyFi™ DNA Polymerase mix (Bioline Inc., Taunton, USA) in a total volume of 20 μl containing 8 pmol of each primer and *c*.50 ng of gDNA. PCR amplicons were purified using the QIAquick PCR purification kit (Qiagen Ltd., Hilden, Germany) following the manufacturerʼs instructions and sequenced directly for both strands using the PCR primers with ABI BigDye chemistry (ABI PerkinElmer, London, UK), alcohol-precipitated, and run on an Applied Biosystems 3730XL DNA analyser at GATC Biotech, Konstanz, Germany.

### 2.3 Phylogenetic analyses

Contiguous sequences were assembled using MEGA v.7 (Kumar et al., 2016) and subjected to a BLASTn search on the NCBI GenBank database. The newly generated and published 28S rDNA sequences were aligned with MAFFT v.7 (Katoh & Standley, 2013) under default gap parameters on the EMBL-EBL bioinformatics web platform (http://www.ebi.ac.uk/Tools/msa/mafft/). The newly generated and published *cox*1 sequences were aligned with MUSCLE implemented in MEGA v. 7 (Kumar et al., 2016) with reference to the amino acid translation, using the echinoderm and flatworm mitochondrial code (translation Table 9; Telford et al., 2000); the alignments included no insertions or deletions. Due to the insufficient variability provided by the amino acids alone, these alignments were analysed solely as nucleotides; first, second and third positions within the included codons were included in these analyses.

Molecular identification and assessment of phylogenetic relationships were achieved using neighbour-joining (NJ) analyses performed on Kimura 2-parameter distances with MEGA v.7 (Kumar et al., 2016). Bayesian inference (BI) analysis was carried out for the 28S rDNA dataset for the Microcotylidae with MrBayes version 3.2.6 using Markov chain Monte Carlo (MCMC) searches on 2 simultaneous runs of 4 chains for 10^7^ generations, sampling trees every 10^3^ generations. The “burn-in” was set for the first 25% of the trees sampled; consensus topology and nodal support were estimated from the remaining 75% of the trees. Prior to the analysis, the best-fitting model of nucleotide substitution was estimated with jModelTest 2.1.4 (Darriba et al., 2012) based on the corrected Akaike (AICc) and the Bayesian information criteria (BIC). This was the general time reversible model with gamma distributed among-site rate variation (GTR+Г). BI analysis was run on XSEDE on the Cipres Science Gateway v. 3.1 (http://www.phylo.org/sub_sections/portal/; Miller et al., 2010). Tree topologies were visualised in FigTree v.1.4.3 (http://tree.bio.ed.ac.uk/software/figtree/.).

The relationships between haplotypes of *S. chrysophrii* were visualised *via* haplotype networks constructed with statistical parsimony analysis using TCS v.1.21 (Clement et al., 2000) under 95% connection limits (Templeton et al., 1992; Clement et al., 2002) and with the median-joining method (Bandelt et al., 1999) using PopART v.1.7 (http://popart.otago.ac.nz/; Leigh & Bryant, 2015).

Pairwise genetic distances (uncorrected p-distance model) were calculated in MEGA v.7.

## 3 Results

### 3.1 Overview of the novel molecular data and morphological assessment

A total of 46 partial sequences (23 for each gene) were generated from 38 isolates of monogeneans found in the four sparid fish hosts sampled off the Algerian coast of the Western Mediterranean (Table 1). The preliminary morphological assessment of the specimens indicated that these belong to three families: Capsalidae [*Encotyllabe* spp., a total of 23 isolates ex *D. vulgaris* (*n* = 4), *S. aurata* (*n* = 5) and *P. bogaraveo* (*n* = 14)]; Microcotylidae [*Atrispinum acarne* Maillard & Noisy, 1979 ex *D. vulgaris* (*n* = 4), *S. chrysophrii* ex *S. aurata* (*n* = 5), *Microcotyle erythrini* van Beneden & Hesse, 1863 ex *P. pagrus* (*n* = 3), and Microcotylidae gen. sp. ex *D. vulgaris* (*n* = 1)]; and Diclidophoridae [*Choricotyle chrysophryi* van Beneden & Hesse, 1863 ex *P. bogaraveo* (*n* = 2)].

**Table 1:** List of the monogenean isolates sampled from *Diplodus vulgaris*, *Pagellus bogaraveo*, *Sparus aurata* and *Pagrus pagrus* off Algeria and used for sequence generation in the present study

Monogenean species	Host species	Isolate	Locality	GenBank ID	
				*cox*1	28S rDNA
Family Microcotylidae Taschenberg, 1879					
*Atrispinum acarne* Maillard & Noisy, 1979	*D. vulgaris*	M40	Off Algiers	OL675203	
	*D. vulgaris*	M50	Off Annaba	OL675204	OL679671
	*D. vulgaris*	M44a	Off Algiers	OL675205	OL679672
	*D. vulgaris*	M44b	Off Algiers		OL679673
*Sparicotyle chrysophrii* (van Beneden & Hesse, 1863) Mamaev, 1984	*S. aurata*	M79	Off Annaba	OL675206	
	*S. aurata*	M82	Off Annaba	OL675207	OL679674
	*S. aurata*	M51	Off Bouzedjar	OL675208	OL679675
	*S. aurata*	M69	Off Bouzedjar	OL675209	
	*S. aurata*	M57	Off Bouzedjar	OL675210	
*Microcotyle erythrini* van Beneden & Hesse, 1863 (*sensu stricto*)	*P. pagrus*	L53	Off Bouzedjar		OL679676
	*P. pagrus*	L54	Off Bouzedjar	OL675211	
	*P. pagrus*	L66	Off Skikda		OL679677
Prostatomicrocotylinae gen. sp.	*D. vulgaris*	M46	Off Algiers	OL675212	
Family Capsalidae Baird, 1853					
*Encotyllabe* sp. 1	*P. bogaraveo*	L112	Off Skikda		OL679678
	*P. bogaraveo*	L113	Off Skikda		OL679679
	*P. bogaraveo*	L116	Off Skikda		OL679680
	*P. bogaraveo*	L262	Off Skikda	OL675214	OL679681
	*P. bogaraveo*	L265	Off Skikda	OL675215	OL679682
	*P. bogaraveo*	L266	Off Skikda	OL675216	OL679683
	*P. bogaraveo*	L283	Off Skikda	OL675217	OL679684
	*P. bogaraveo*	L126	Off Skikda		OL679685
	*P. bogaraveo*	L248	Off Algiers	OL675218	
	*P. bogaraveo*	L94	Off Bouzedjar	OL675219	
	*P. bogaraveo*	L123	Off Bouzedjar	OL675220	
	*P. bogaraveo*	L106	Off Skikda	OL675221	
*Encotyllabe* sp. 2	*D. vulgaris*	M36	Off Algiers		OL679686
	*D. vulgaris*	M43	Off Algiers		OL679687
	*D. vulgaris*	M20	Off Bouzedjar		OL679688
	*S. aurata*	M80	Off Annaba		OL679689
	*S. aurata*	M56	Off Bouzedjar	OL675222	OL679690
	*S. aurata*	M60a	Off Bouzedjar		OL679691
	*S. aurata*	M60b	Off Bouzedjar		OL679692
*Encotyllabe* sp.	*P. bogaraveo*	L100	Off Bouzedjar	OL675223	
	*P. bogaraveo*	L109	Off Skikda	OL675224	
	*D. vulgaris*	M17	Off Bouzedjar	OL675225	
	*S. aurata*	M68	Off Bouzedjar	OL675226	
Family Diclidophoridae Cerfontaine, 1895					
*Choricotyle chrysophryi* van Beneden & Hesse, 1863	*P. bogaraveo*	L244	Off Algiers	OL675213	
	*P. bogaraveo*	L228	Off Bouzedjar		OL679693

Comparative sequence and phylogenetic analyses carried out here confirmed the identification based on morphology for the diclidophorid *C. chrysophryi* and the microcotylids except for Microcotylidae gen. sp. ex *D. vulgaris* and indicated the presence of two putative species of *Encotyllabe* Diesing, 1850.

The morphology of the specimens of the three species previously recorded in sparids (*C. chrysophryi*, *A. acarne* and *S. chrysophrii*) agreed with the redescription of Llewellyn (1941), the original description of Maillard & Noisy (1979) and the redescription of Euzet & Noisy (1979), respectively, as well as with the data provided by Radujković & Euzet (1989) (*C*. *chrysophryi* and *S. chrysophrii*). Morphological identification of species of *Microcotyle* is not straightforward because of the morphological homogeneity of the species within the *M. erythrini* species complex (see Bouguerche et al., 2019a, b; Víllora-Montero et al., 2020). Therefore, identification of the present specimens of *Microcotyle* was based on the morphological and molecular characterisation of *M. erythrini* (*s.s.*) of Víllora-Montero et al. (2020).

Unfortunately, the condition of the single specimen ex *D. vulgaris* provisionally identified as Microcotylidae gen. sp. did not allow examination of its morphology in detail. However, this isolate fell within the clade representing the subfamily Prostatomicrocotylinae with strong support in the analysis of the *cox*1 sequences. Regarding the specimens of *Encotyllabe* sequenced here, it is worth noting that these were not assigned *a priory* to one of the two forms; this separation was based on the consistent nucleotide differences and clustering of the specimens based on the more conservative 28S rRNA gene. The two forms delineated by the 28S analysis, exhibit morphological differences, the most apparent being the shape and size of the large hamuli. However, detailed morphological description of the present material of *Encotyllabe* spp. requires a revision of the genus and will be published elsewhere.

### 3.2 Family Microcotylidae Taschenberg, 1879

#### 3.2.1 Analysis 1: cox1 dataset

**Fig. 1:**
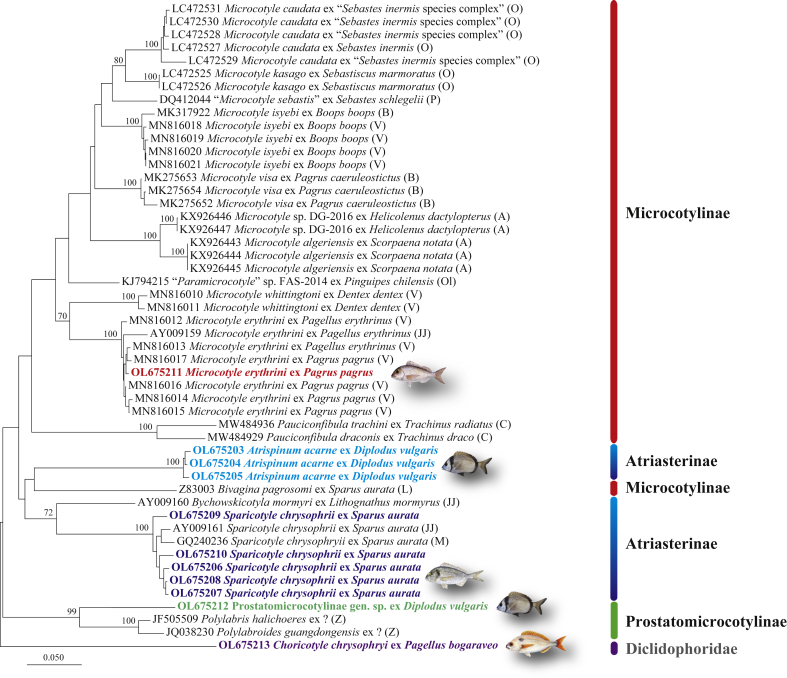
Neighbour-joining phylogram from analysis of the *cox*1 alignment (304 nt) for the Microcotylidae including 11 newly generated sequences and 39 sequences from GenBank. Outgroup: *Choricotyle chrysophryi* (Diclidophoridae). Only nodal support values > 70% are shown. The scale-bar indicates the expected number of substitutions per site. Sequence identification and hosts are indicated as in GenBank, followed by a letter: A, Ayadi et al. (2017); Az, Azizi et al. (2021); B, Bouguerche et al. (2019a, b); JJ, Jovelin & Justine (2001); L, Littlewood et al. (1997); M, Mladineo et al. (2009); O, Ono et al. (2020); Ol, Oliva et al. (2014); P, Park et al. (2007); V, Víllora-Montero et al. (2020); Z, Zhang et al. (2011) (see Table 1 and Supplementary Table S1 for details).

The alignment of the *cox*1 dataset for the Microcotylidae (304 nt) comprised 11 newly generated sequences (one for the outgroup, *C. chrysophryi*; see Table 1) and 39 sequences retrieved from GenBank (see Supplementary Table S1 for details). The neighbour-joining tree (Fig. 1) revealed the monophyly of the genus *Microcotyle* van Beneden & Hesse, 1863 and the subfamily Prostatomicrocotylinae Yamaguti, 1963. However, a species of the only other genus representative of the subfamily Microcotylinae Taschenberg, 1879 in this analysis, *Bivagina pagrosomi* (Murray, 1931) Dillon & Hargis, 1965, clustered with *A. acarne* within the clade representing the subfamily Atriasterinae Maillard & Noisy, 1979 (*Atrispinum* Euzet & Maillard, 1974 + *Bychowskicotyla* Unnithan, 1971 + *Sparicotyle* Mamaev, 1984).

The *cox*1 analysis provided molecular confirmation for the identification of *M. erythrini* (*sensu stricto*) as defined by Víllora-Montero et al. (2020) [one isolate ex *P. pagrus* clustering with a sequence ex *P. erythrinus* (GenBank: AY009159) from off France by Jovelin & Justine (2001) and 6 sequences ex *P. pagrus* and *P. erythrinus* from off Spain by Víllora-Montero et al., 2020] and *S. chrysophrii* [five isolates ex *S. aurata* clustering with sequences ex *S. aurata* from off France (Jovelin & Justine, 2001) and fish farms in the Adriatic Sea (Mladineo et al., 2009)].

The isolates of *S. chrysophrii* from off Algeria represented 5 unique novel haplotypes. Additionally, one isolate ex *D. vulgaris* provisionally identified as Microcotylidae gen. sp. fell within a strongly supported clade representing the subfamily Prostatomicrocotylinae and comprising the sequences for *Polylabris halichoeres* Wang & Zhang, 1998 and *Polylabroides guangdongensis* Zhang & Yang, 2000. There were no *cox*1 sequences for *A. acarne* on GenBank but two of the three isolates sequenced for *cox*1 were also sequenced for 28S (see below); thus, our study provides the first *cox*1 sequences for *A. acarne*.

**Fig. 2:**
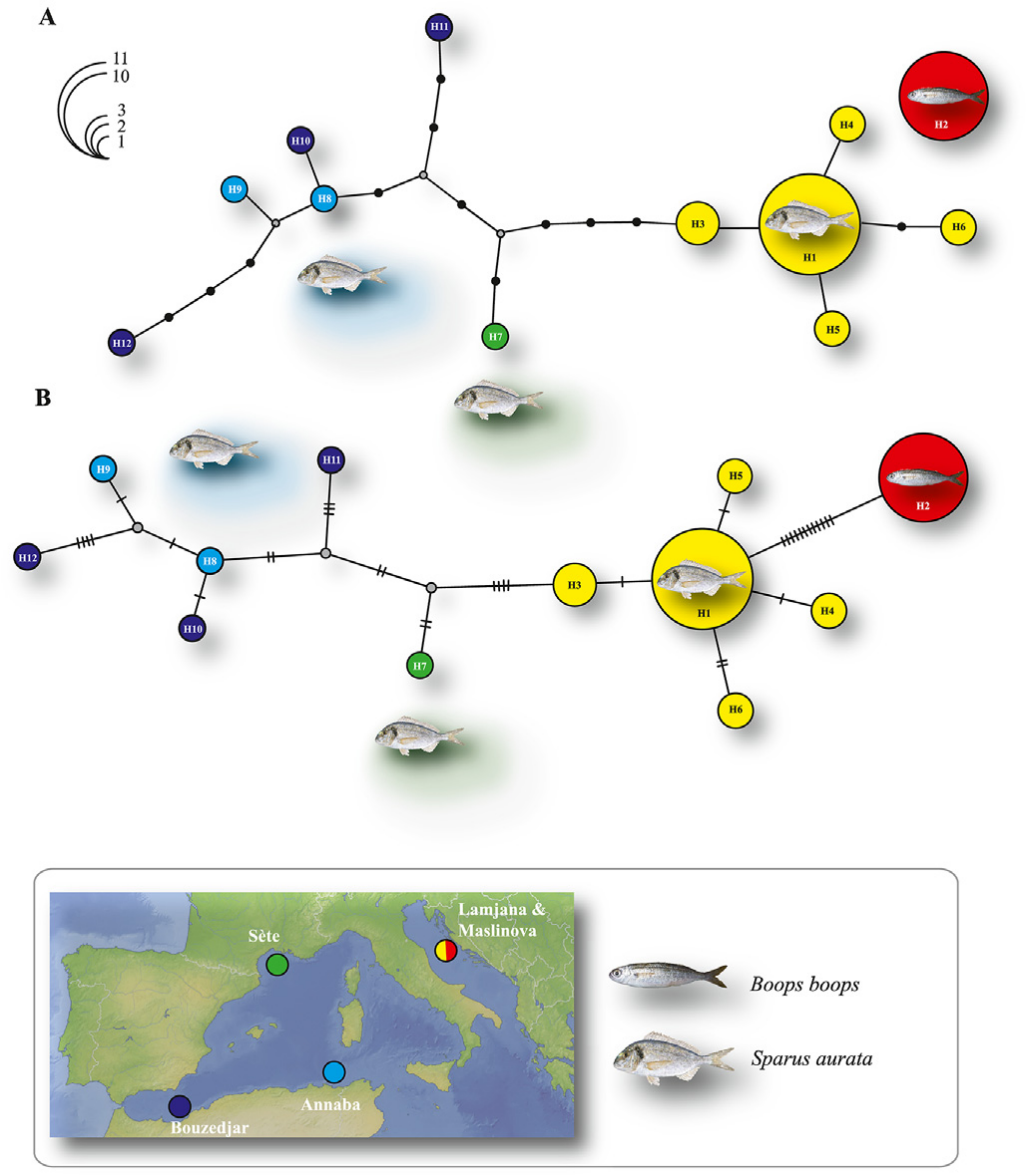
Haplotype networks for *Sparicotyle chrysophrii* from the Mediterranean built using 36 partial mitochondrial *cox*1 sequences (see Table 1 and Supplementary Table S2 for details). **A** Statistical parsimony network constructed with TCS 1.21 software under 95% connection limits. The connecting lines indicate the maximum number of steps connecting parsimoniously two haplotypes. The small black circles represent alternative unsampled haplotypes. **B** Comparative median-joining network built with PopART v.1.7. The hatch marks on the connecting lines indicate the number of the mutational steps among the haplotypes. **A**-**B** Pie chart sizes are proportional to haplotype frequency; haplotype frequency scale is indicated by colourless semicircles. Circle colours indicate the locations of origin for the distinct haplotypes as follows: yellow/red: Adriatic Sea, off Croatia; green, off Sète, France; dark blue, off Bouzedjar, Algeria; light blue, off Annaba, Algeria. *Abbreviations*: H1–H12, haplotype IDs.

Mitochondrial *cox*1 haplotype networks were constructed for the important fish pathogen in sparid aquaculture *S*. *chrysophrii* based on the newly generated and published sequences from sparid fishes in the Central and Western Mediterranean (Jovelin & Justine, 2001; Mladineo et al., 2009) (see Supplementary Table S2 for details). The aligned *cox*1 dataset for *S*. *chrysophrii* comprised 304 nt positions. A total of 12 unique haplotypes was identified in the 36 individual sequences included in this alignment: six from cage-reared *S. aurata* and wild *Boops boops* associated with these cages in the Central Mediterranean (Adriatic Sea, see Mladineo et al., 2009); and six (all singletons) from *S. aurata* sampled in the Western Mediterranean off Sète (Jovelin & Justine, 2001) and Algeria (present study) (Supplementary Table S2). As illustrated in Fig. 2, there is a significant differentiation between the haplotypes of *S*. *chrysophrii* from *S. aurata* in the Central and Western Mediterranean (genetic divergence of 2.3–3.9% compared with 0.3–0.7% for the haplotypes from the Central Mediterranean alone) and that the haplotype from *B. boops* may represent a cryptic species (genetic divergence of 3.9–7.9% compared with 0.3–3.9% for the haplotypes from *S. aurata*).

#### 3.2.2 Analysis 2: molecular phylogeny of the Microcotylidae based on 28S rDNA

The alignment of the 28S rDNA dataset for the family Microcotylidae (886 nt) comprised 8 newly generated sequences (one used as the outgroup, *C. chrysophryi*; see Table 1) and 37 sequences for representatives of 19 genera retrieved from GenBank (see Supplementary Table S1 for details). The sequences for two species of the Metamicrocotylinae Yamaguti, 1963, i.e. *Metamicrocotyla mugilis* Yamaguti, 1968 (GenBank: AF131722; 357 nt) and *Metamicrocotyla cephalus* (Azim, 1939) Hargis, 1954 (GenBank: AF131720; 350 nt) (Mollaret et al., 2000), are very short and were not included in the analysis.

**Fig. 3:**
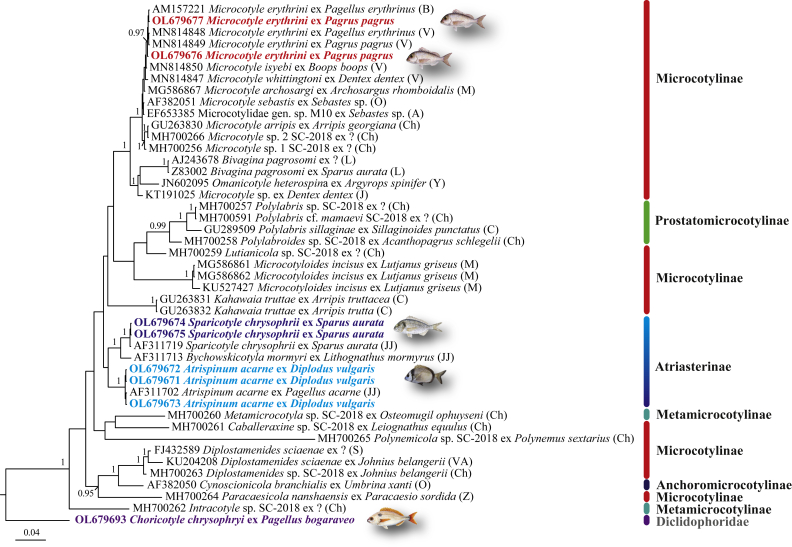
Phylogram from Bayesian inference analysis of the 28S rDNA sequence alignment (886 nt) for the Microcotylidae including 8 newly generated sequences and 36 sequences from GenBank. Outgroup: *Choricotyle chrysophryi* (Diclidophoridae). Only nodal support values > 0.95 are shown. The scale-bar indicates the expected number of substitutions per site. Sequence identification and hosts are shown as on GenBank except for KT191025 Microcotylidae sp. JWJ-2015 (identification as in the published paper), followed by a letter: A, Aiken et al. (2007); B, Badets et al. (2011); C, Catalano et al. (2010); Ch, Chou (unpublished); J, Jun (2015); JJ, Jovelin & Justine (2001); M, Mendoza-Franco et al. (2018); L, Littlewood et al. (1997, 1999); O, Olson & Littlewood (2002); S, Su (unpublished); V, Víllora-Montero et al. (2020); VA, Verma & Agrawal (unpublished); Y, Yoon et al. (2013); Z, Zhou et al. (2020) (see Supplementary Table S1 for details).

The BI phylogeny based on this dataset (Fig. 3) represents a poorly resolved hypothesis for the Microcotylidae and the groupings at the subfamilial level of the representatives for which molecular data are available. The most abundantly sampled microcotylid subfamily, the Microcotylinae (represented by species of 10 out of 30 genera), was recovered as polyphyletic and only one subfamily, the Prostatomicrocotylinae (represented by species of 2 out of 5 genera, *Polylabris* Euzet & Cauwet, 1967 and *Polylabroides* Mamaev & Parukhin, 1976), received strong statistical support (Fig. 3). The Atriasterinae, represented by 3 species of the genera *Atrispinum* Euzet & Maillard, 1974, *Bychowskicotyla* Unnithan, 1971 and *Sparicotyle* Mamaev, 1984, was resolved in a separate but poorly supported clade (posterior probability < 0.95%). Finally, there was a strong association between *Diplostamenides* spp. (Microcotylinae) and *Cynoscionicola branchialis* (Anchoromicrocotylinae Bravo-Hollis, 1981) and for the sister-group relationship of *Paracaesicola nanshaensis* Zhou, Li, Liu, Ding & Yuan, 2020 (Microcotylinae) joining this clade (Fig. 3).

At the generic level, seven species of *Microcotyle* formed a strongly supported clade, sister to a clade representing *Omanicotyle* Yoon, Al-Jufaili, Freeman, Bron, Paladini & Shinn, 2013 and *Bivagina* Yamaguti, 1963. Two otherwise unpublished sequences by Chou (*Microcotyle* spp. 1 and 2, no host and locality data available) and a sequence (GenBank: EF653385 Microcotylidae gen. sp. M10) for an unidentified microcotylid ex *Sebastes* sp. off UK published by Aiken et al. (2007) clustered within the clade of *Microcotyle* spp. and thus clearly represent sequences for three distinct species of *Microcotyle*. However, the sequence KT191025 reported by Jun (2015) as *Microcotyle* sp. ex *Dentex dentex* (L.) from the Eastern Mediterranean (off Crete) was strongly associated with *B. pagrosomi* and *Omanicotyle heterospina* Yoon, Al-Jufaili, Freeman, Bron, Paladini & Shinn, 2013 and thus does not belong to *Microcotyle* (Fig. 3).

The newly generated sequences from isolates parasitic in the Mediterranean sparids off Algeria clustered with a strong support with: (i) a sequence for *A. acarne* ex *Pagellus acarne* (GenBank: AF311702) from off France by Jovelin & Justine (2001) (3 isolates ex *D. vulgaris*); (ii) a sequence for *S. chrysophrii* ex *S. aurata* (GenBank: AF311719) from off France by Jovelin & Justine (2001) (2 isolates ex *S. aurata*); and (iii) sequences for *M. erythrini* (*s.s.*) ex *P. erythrinus* and *P. pagrus* from off France and Spain by Badets et al. (2011) and Víllora-Montero et al. (2020) (Fig. 3).

### 3.3 Family Capsalidae Baird, 1853

**Fig. 4:**
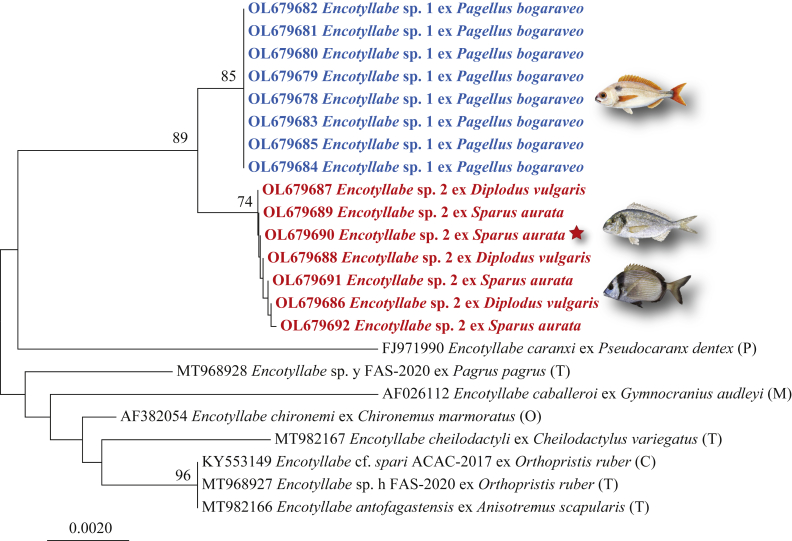
Unrooted neighbour-joining phylogram for the 28S rDNA alignment (771 nt) for *Encotyllabe* spp. including 15 newly generated sequences and 8 sequences from GenBank. Only nodal support values > 70% are shown. The scale-bar indicates the expected number of substitutions per site. Sequence identification and hosts are indicated as on GenBank, followed by a letter: C, Camargo et al. (2017); M, Mollaret et al. (1997); P, Perkins et al. (2009); O, Olson & Littlewood (2002); T, Taborda et al. (unpublished). The star indicates the single isolate of *Encotyllabe* sp. 2 for which a *cox*1 sequence was generated (indicated with a red star in Fig. 5) (see Table 1 and Supplementary Table S1 for details).

The 15 newly generated 28S rDNA sequences (771 nt) from specimens of *Encotyllabe* spp. formed two strongly supported monophyletic clades tentatively labelled *Encotyllabe* sp. 1 ex *P. bogaraveo* and *Encotyllabe* sp. 2 ex *D*. *vulgaris* and *S*. *aurata* (Fig. 4). The sequences of these two forms differed at 2 nt positions and collectively differed from the available sequences for the remaining congeneric species at 4–12 nt positions. Additionally, the sequences for *E*. cf. *spari* ACAC-2017 (GenBank: KY553149; Camargo et al., 2017), *Encotyllabe* sp. h FAS-2020 isolate F44 (GenBank: MT968927; unpublished) and *Encotyllabe antofagastensis* Sepúlveda, González & Oliva, 2014 isolate F1 (GenBank: MT982166; Sepúlveda et al., 2014) are identical. It is also worth noting that the only available sequence for *Encotyllabe caranxi* Lebedev, 1967 (GenBank: FJ971990) is very short (351 nt).

**Fig. 5:**
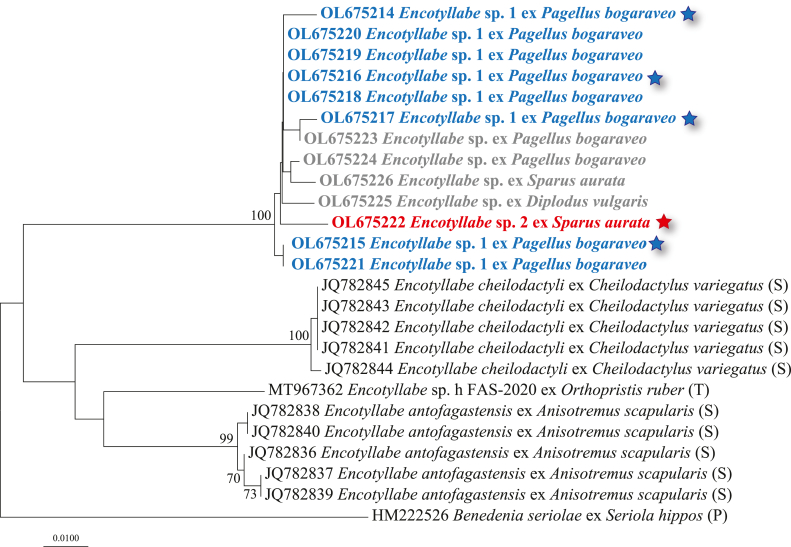
Neighbour-joining phylogram from analysis of the *cox*1 alignment (269 nt) for *Encotyllabe* spp. including 13 newly generated sequences and 11 sequences from GenBank. Outgroup: *Benedenia seriolae* (Capsalidae). Only nodal support values > 70% are shown. The scale-bar indicates the expected number of substitutions per site. Sequence identification and hosts are indicated as in GenBank, followed by a letter: S, Sepúlveda et al. (2014), P, Perkins et al. (2010); T, Taborda et al. (unpublished data). Stars indicate the isolates of *Encotyllabe* for which 28S rDNA sequences were generated (see Table 1 and Supplementary Table S1 for details).

By contrast, in the phylogeny based on the *cox*1 dataset (269 nt) for *Encotyllabe* spp. comprising 13 newly generated sequences and 12 sequences retrieved from GenBank (outgroup: *Benedenia seriolae* (Yamaguti, 1934) Meserve, 1938), all novel sequences formed a single strongly supported monophyletic clade (Fig. 5). However, due to sequencing problems only a single isolate of *Encotyllabe* sp. 2 ex *S*. *aurata* was sequenced for both genes (indicated with a red star in Fig. 5). Of the eight isolates for which we were not able to generate 28S rDNA sequences, three (OL675218-OL675220) were identical with a *cox*1 sequence for *Encotyllabe* sp. 1 (OL675216), one (OL675221) was identical with another sequence for *Encotyllabe* sp. 1 (OL675215) and four could not be assigned to one of the two forms of *Encotyllabe* based on the phylogenetic results. The overall range of genetic divergence for the *cox*1 gene in the newly collected material of *Encotyllabe* was low (0–1.9%; 0–5 nt) but the upper limit was slightly higher than the upper limits of the intraspecific ranges of 0.7% (2 nt) and 0.4% (1 nt) for *E. antofagastensis* and *Encotyllabe cheilodactyli* Sepúlveda, González & Oliva, 2014, respectively. *Encotyllabe* sp. 1 and *Encotyllabe* sp. 2 differed at 3–5 nt positions (1.1–1.9%), values above the known intraspecific divergence for *cox*1 in *Encotyllabe*.

### 3.4 Family Diclidophoridae Cerfontaine, 1895

Our study provides the first 28S rDNA and *cox*1 sequences for *C. chrysophryi* ex *P. bogaraveo*; these were used as the outgroups in the phylogenetic analyses. The newly generated 28S sequence (876 nt) differed from the only 28S sequence for *C. chrysophryi* available on GenBank (AF311705; ex *P. acarne* off France, see Jovelin & Justine, 2001) at 2 nucleotide positions (overlap 505 nt).

A comparison of the newly generated *cox*1 sequence for *C. chrysophryi* (alignment length of 304 nt) with the sequence for *C.* cf. *chrysophryi* ex *P. acarne* off France by Jovelin & Justine (2001) and the two sequences of *C.* cf. *chrysophryi* (as *C. chrysophryi* on GenBank) ex *P. acarne* off Algeria published recently by Bouguerche et al. (2021) revealed a wide range of genetic divergence: (i) very low (0.7%, 2 nt; our sequence *vs* MZ127222 by Bouguerche et al. (2021)); (ii) intermediate (6.3–7.0%, 19–21 nt; our sequence and MZ127222 *vs* AY009165 by Jovelin & Justine (2001)); and (iii) rather high (9.5–15.0%, 28–44 nt, the three sequences listed above *vs* the second sequence (MZ127216) by Bouguerche et al. (2021)). It is worth noting that the sequences AY009165 by Jovelin & Justine (2001) and MZ127216 by Bouguerche et al. (2021) have some ambiguously coded nucleotide positions (3 and 8, respectively) thus increasing to a degree the divergence levels. Nevertheless, the genetic distances between the divergent sequence MZ127216 and the remaining sequences for *C. chrysophryi* seem to fall above the levels of intraspecific divergence. There are three sequences available on GenBank that provide data for congeneric comparisons, i.e. *C*. *australiensis* ex *Chrysophrys auratus* (GenBank: MT783687) off Australia recently re-described by Hossen et al. (2020). Comparative sequence analysis revealed levels of interspecific divergence of 18.8–22.3% between the Mediterranean and Australian species of *Choricotyle.*

## 4 Discussion

To the best of our knowledge this is the first molecular assessment of polyopisthocotyleans in sparids which is based on a focused systematic sampling of a range of hosts and in diverse coastal environments of the Western Mediterranean. Overall, our study expands the 28S rDNA and *cox*1 sequence databases with 46 sequences for seven species of three monogenean families (Capsalidae, Microcotylidae and Diclidophoridae). Comparative sequence analyses and phylogenetic reconstructions confirmed the morphological identification of four monogenean species (*A. acarne*, *C. chrysophryi*, *M. erythrini* (*s.s.*) and *S. chrysophrii*), helped assign the microcotylid from *D. vulgaris* provisionally identified as Microcotylidae gen. sp. to the subfamily Prostatomicrocotylinae and revealed that the capsalid specimens sequenced may belong to two putative species. Our study provides (i) the first molecular data for the Capsalidae in the Mediterranean; (ii) the first record of a member of the Prostatomicrocotylinae in the Mediterranean and in a sparid fish (*D. vulgaris*); (iii) the first *cox*1 sequences for *A. acarne*; (iv) the second record of *M. erythrini* (*s.s.*) from *P. pagrus*; and (v) the second confirmed by molecular data record of *S. chrysophrii* in wild populations of *S. aurata.*

The recent accumulation of 28S rDNA sequences for the Microcotylidae offers the opportunity for an updated phylogenetic hypothesis for this monogenean group. The first attempt to assess the relationships of the Microcotylidae based on 28S rDNA data was included within a wider reconstruction of the phylogenies of the Monopisthocotylea and Polyopisthocotylea (see Mollaret et al., 2000). These authors included sequences for representatives of five microcotylid genera, i.e. *A. acarne* (GenBank: AF131713), *Polylabris heterodus* (Lebedev & Parukhin, 1969) Mollaret, Jamieson & Justine, 2000 (GenBank: AF131716), *Solostamenides mugilis* (Vogt, 1879) Unnithan, 1971 (as *Microcotyle mugilis*, GenBank: AF131722), *M. cephalus* (Azim, 1939) Hargis, 1954 (GenBank: AF131720) and *Bivagina pagrosomi* (GenBank: Z83002) and suggested non-monophyly of the Microcotylidae (see Mollaret et al., 2000).

Jovelin & Justine (2001) inferred the relationships within the Polyopisthocotylea, and especially, between the terminal groups (Gastrocotylinea, Discocotylinea and Microcotylinea), based on partial (domain D2) 28S rDNA sequences. These authors provided new 28S sequences for representatives of four microcotylid genera, i.e. *A. acarne* (GenBank: AF311702), *C.* cf. *chrysophryi* (GenBank: AF311705), *M. erythrini* (GenBank: AF311712) and *S. chrysophrii* (GenBank: AF311719); they also provided novel *cox*1 sequences for the latter three species (GenBank: AY009165, AY009159 and AY009161, respectively). Jovelin & Justine (2001) found the Microcotylidae to be monophyletic but the relationships within the family were poorly supported; a single clade (*B*. *pagrosomi* + *M. erythrini*) was well supported.

The present molecular analyses of the relationships within the Microcotylidae are based on a markedly expanded taxa representation at all levels, i.e. subfamily, genus, species. The phylogeny based on the 28S rRNA gene includes five of the seven subfamilies of the Microcotylidae, i.e. Anchoromicrocotylinae, Atriasterinae Maillard & Noisy, 1979, Metamicrocotylinae Yamaguti, 1963, Microcotylinae and Prostatomicrocotylinae, which are represented by one to eight genera (19 genera in total). The largest microcotylid subfamily, the Microcotylinae, is the most densely sampled (represented by species of 10 out of 30 genera). The phylogeny based on the *cox*1 gene includes fewer taxa, representative for three subfamilies: Microcotylinae (2 genera, 12 species); Atriasterinae (3 genera, 3 species) and Prostatomicrocotylinae (2 genera, 3 species). Overall, 11 of the species included in the phylogenetic analyses represented isolates originating from the Western Mediterranean (Table 1 and Supplementary Table S1). These are representative for six genera thus comprising nearly 50% of the known microcotylid diversity in the Mediterranean (29 species of 14 genera, see Gibson et al., 2005; Ayadi et al., 2017; Bouguerche et al., 2019a, b; Víllora-Montero et al., 2020; Azizi et al., 2021). The fact that our large-scale intensive sampling off Algeria did not increase the number of species indicates that a substantial proportion of microcotylid diversity in the Western Mediterranean has already been discovered and is represented in our phylogenetic hypotheses.

The molecular phylogeny based on 28S rDNA data (Fig. 3) provided a poorly resolved hypothesis for the relationships at the subfamilial level and demonstrated the non-monophyly of the most widely represented subfamily, the Microcotylidae, with a main strongly supported clade containing *Microcotyle* + *Omanicotyle* + *Bivagina* and several members positioned in six distinct clades (*Caballeraxine* Lebedev, 1972; *Diplostamenides* Unnithan, 1971; *Kahawaia* Lebedev, 1969; *Lutianicola* Lebedev, 1970; *Microcotyloides* Fujii, 1940; *Paracaesicola* Zhou, Li, Liu, Ding & Yuan, 2020; and *Polynemicola* Unnithan, 1971).

Both 28S rDNA and *cox*1 phylogenies provided strong support for the Prostatomicrocotylinae represented by different species of *Polylabris* and *Polylabroides* (Figs. 1 and 3). However, the two datasets yielded contradictory results for the Atriasterinae. This family represented by three out of six genera (*Atrispinum*, *Sparicotyle* and *Bychowskicotyla*), was also recovered as monophyletic albeit with poor support in the 28S phylogeny but the only available sequence for *Bivagina pagrosomi* (GenBank: Z83003) clustered with sequences for *A. acarne* within the clade of the Atriasterinae in the *cox*1 analysis. It is difficult to interpret this result as the *cox*1 sequence for this isolate has only been used as the outgroup in previous analyses (Littlewood et al., 1997; Bouguerche et al., 2019a, b). The identification of the host (as “*S. aurata*”) for this isolate is also ambiguous as the occurrence of this sparid off Australia is considered questionable (Froese & Pauly, 2021). A plausible explanation would seem to be that *Bivagina pagrosomi* (GenBank: Z83003) represents a misidentification of the only species of *Atriaster* Lebedev & Parukhin, 1969 reported from a sparid host off Australia, i.e. *Atriaster spinifer* Mamaev & Parukhin, 1975 ex *Argyrops spinifer* (Forsskål).

Overall, although the present phylogenetic analyses represent a significant step towards a more comprehensive phylogenetic hypothesis of the Microcotylidae, the non-monophyly of both the largest and one of the smallest subfamilies (Microcotylinae and Metamicrocotylinae, respectively), clearly indicate that the current subfamily structure of the Microcotylidae may not be representative of the phylogenetic relationships of its genera. In his taxonomic revision of the family, Mamaev (1986) indicated that “… in particular, the subfamily Microcotylinae is too heterogeneous, and possibly it will be divided into 2–3 subfamilies”. The pattern observed in the 28S rDNA phylogeny tends to support this hypothesis as indicated by the disparate placement of *Caballeraxine*, *Diplostamenides*, *Microcotyloides*, *Paracaesicola* and *Polynemicola.* However, the taxonomic diversity of this subfamily is still too poorly represented in the molecular phylogeny due to the lack of sequence data for 20 genera (67%): *Diplasiocotyle* Sandars, 1944; *Pauciconfibula* Dillon & Hargis, 1965; *Pseudoaspinatrium* Mamaev, 1986; *Pseudobivagina* Mamaev, 1986; *Sciaenacotyle* Mamaev, 1989; *Solostamenides* Unnithan, 1971; plus 14 monotypic genera (*Atriostella* Unnithan, 1971; *Gamacallum* Unnithan, 1971; *Jaliscia* Mamaev & Egorova, 1977; *Magniexcipula* Bravo-Hollis, 1981; *Monomacracanthus* Mamaev, 1976; *Neobivagina* Dillon & Hargis, 1965; *Paramicrocotyloides* Rohde, 1978; *Paranaella* Kohn, Baptista-Farias & Cohen, 2000; *Polymicrocotyle* Lamothe-Argumedo, 1967; *Pseudoneobivagina* Mamaev, 1986; *Sebasticotyle* Mamaev & Egorova, 1977; *Vulvostella* Unnithan, 1971; *Yogendrotrema* Kumar & Agarwal, 1983; and *Neobivaginopsis* Villalba, 1987).

In a similar vein, further denser and focused sampling is required before attempting a more comprehensive phylogeny that would reflect adequately the taxonomic diversity of the Microcotylidae. The following taxa require further exploration in a molecular phylogeny: (i) the unsampled Syncoelicotylinae Mamaev & Zubchenko, 1978 (with 3 genera: *Syncoelicotyle* Mamaev & Zubchenko, 1978; *Syncoelicotyloides* Mamaev & Brashovian, 1989; and *Tinrovia* Mamaev, 1987) and the monotypic Prosomicrocotylinae Yamaguti, 1963 (*Prosomicrocotyla* Yamaguti, 1958); and (ii) the insufficiently sampled Atriasterinae (no sequences available for the genera *Atriaster*, *Serranicotyle* Maillard, Euzet & Silan, 1988, and *Pagellicotyle* Mamaev, 1984) and Prostatomicrocotylinae (no sequences available for the genera *Aspinatrium* Yamaguti, 1963, *Grandicotyle* Lebedev, 1976, and *Tonkinaxine* Lebedev, Parukhin & Roitman, 1970).

*Microcotyle*, the most species-rich genus within the Microcotylinae (69 spp.; WoRMS, 2021), was most densely sequenced for both 28S and *cox*1 markers (9 and 10 spp., respectively) and was recovered as monophyletic in both analyses. The analysis based on 28S rDNA data indicates the presence of three distinct yet unidentified species of *Microcotyle* (*Microcotyle* sp. 1, *Microcotyle* sp. 2 and Microcotylidae gen. sp.) and that a species of another microcotylid genus ex *Dentex dentex* has been misidentified as *Microcotyle*. It is worth noting that the only species of *Microcotyle* parasitising this host in the Mediterranean, *M. whittingtoni* Víllora‑Montero, Pérez‑del‑Olmo, Georgieva, Raga & Montero, 2020, has been described just recently (Víllora-Montero et al., 2020); the 28S rDNA sequence provided by these authors clearly belongs to the monophyletic clade of *Microcotyle* (Fig. 3). The analysis based on *cox*1 data confirmed the synonymisation of *Paramicrocotyle* Caballero & Bravo-Hollis, 1972 with *Microcotyle* by Mamaev (1986) and Bouguerche et al. (2019b) (see Bouguerche et al., 2019b for an earlier *cox*1 analysis and a detailed discussion).

Although the important fish pathogen *S. chrysophrii* has been recorded in wild populations of *S. aurata* (e.g. Euzet & Audouin, 1959; Radujkovic & Euzet, 1989) and *Diplodus puntazzo* (Walbaum) (see Sánchez-García et al., 2015), most of the genetic data come from cage-reared *S. aurata* in the Adriatic (Mladineo et al., 2009) (partial *cox*1 gene, 5 haplotypes, see Supplementary Table S2). Our study revealed a similar genetic diversity (6 haplotypes) based on a very limited sampling of wild populations of *S. aurata* in the Western Mediterranean. This, coupled with the significant differentiation between the haplotypes of the Adriatic and Western Mediterranean populations of *S. chrysophrii* (Fig. 2), indicates a much greater genetic diversity of this species in the wild populations of its sparid hosts (*S. aurata* and *D. puntazzo*) in the Western Mediterranean; wider sampling in this region is needed to test this hypothesis that might have serious repercussions for sea-cage sparid aquaculture in this region. Finally, the very divergent haplotype from *B. boops* may represent a cryptic species of *Sparicotyle* pending additional morphological and molecular study.

Our study provides the first 28S rDNA and *cox*1 sequences for species of *Encotyllabe* in the Mediterranean and adds to the rather scarce molecular data for this genus worldwide. The genus currently contains 24 species (WoRMS, 2021). Of these, only three have been recorded in sparid and labrid fishes in the Mediterranean: *Encotyllabe nordmanni* Diesing, 1850 ex *Sparus brama* Bonnaterre and *Chromis chromis* (L.); *Encotyllabe paronae* Monticelli, 1907 ex *Symphodus tinca* (L.); and *Encotyllabe vallei* Monticelli, 1907 ex *D. puntazzo* and *S. aurata* (see Euzet et al., 1993). However, the original descriptions of these species are too brief and insufficient for species differentiation/identification and there is just one study (Radujkovich & Euzet, 1989) providing some morphological data for a single specimen of *E. vallei*. Thus, elucidating the actual diversity of *Encotyllabe* in the Mediterranean would require detailed morphological studies, preferably in association with molecular data.

Comparative sequence analyses of the Microcotylidae showed that both partial 28S and *cox*1 genes can be successfully used for species identification. However, the number of sequences available for representatives of the family for both loci is still very low in spite of the recent surge in the use of *cox*1 DNA data for monogenean species discovery and identification in the Western Mediterranean (e.g. Chaabane et al., 2016a, b; Kheddam et al., 2016; Ayadi et al., 2017; Bouguerche et al., 2019a, b, Víllora-Montero et al., 2020; Bouguerche et al., 2021).

Although the use of the short sequences of the barcoding *cox*1 gene may substantially facilitate and accelerate identification of previously characterised species especially in large-scale inventories and aquaculture settings, amplification success is considered to represent the major limitation to a wider use of *cox*1 barcoding in monogeneans (Vanhove et al., 2013). Whilst we have been successful in amplifying the *cox*1 fragment for the microcotylids, this was not the case with the specimens of *Encotyllabe*; the amplified fragment was short, and amplification failed for most isolates of *Encotyllabe* sp. 2. The position of the single successfully sequenced isolate in the *cox*1 phylogeny did not support the distinct status of this putative species. This scenario may occur in other monogenean groups so that for species distinction using *cox*1 it is necessary to generate more sequences per taxon and rely on the detection of reciprocally monophyletic lineages in phylogenies. Further, the available data are insufficient to assess the intra- and interspecific genetic variation for most polyopisthocotylean genera. Clearly vast additional taxon sampling and preferably sequencing of both 28S rRNA and *cox*1 genes would help advance species discovery and identification of the monogenean groups studied.

## 5 Conclusions

Our focused systematic sampling of monogeneans in four sparid hosts from the Western Mediterranean off Algeria expanded the 28S rDNA and *cox*1 sequence databases with 46 sequences for seven species of the families Microcotylidae, Capsalidae and Diclidophoridae. Comparative sequence and phylogenetic analyses confirmed species identification based on morphology and helped clarify the status of some species of *Microcotyle* and *Choricotyle.* The first phylogenetic hypotheses for the family Microcotylidae provided here, revealed the monophyly of the subfamily Prostatomicrocotylinae and the genus *Microcotyle* but the relationships among the subfamilies were still largely unresolved with the largest and best represented subfamily Microcotylinae being polyphyletic. We highlight that a thorough taxon-sampling approach is required to increase the accuracy of phylogenetic reconstruction of the relationships of the diverse polyopisthocotylean family Microcotylidae.

## Funding

This study was partially funded by the Czech Science Foundation (projects P505/10/1562 and ECIP P505/12/G112) (AK, SG), by the Laboratory Reseau de Surveillance Environmental (LRSE), University Oran 1 Algeria (n°10, W0911505) (DM) and the Generalitat Valenciana, Spain (GV/2019/143) (SG). SG benefited from the postdoctoral fellowship programme Juan de la Cierva Formación of the Ministry of Science, Innovation and Universities (MICINN), Spain (FJCI-2016-29535).

## Ethical approval

Not applicable. Fishes were purchased from the local fish markets.

## CRediT author statement

Aneta Kostadinova and Douniazed Marzoug conceived and designed the study. Lamia Lablack and Mohammed Rima collected the samples in the field and performed the laboratory analyses, preliminary identification, and morphological characterisation of the isolates. Simona Georgieva carried out sequencing and phylogenetic analyses. Lamia Lablack and Simona Georgieva wrote the first draft of the manuscript. Aneta Kostadinova coordinated the project and helped draft the manuscript. All authors critically reviewed the manuscript, and read and approved the final manuscript.

## Data availability

The newly generated sequences are deposited in the GenBank database under the accession numbers OL675203-OL675226 (*cox*1 gene) and OL679671-OL679693 (28S rRNA gene). The voucher specimens are deposited at the Helminthological Collection of the Institute of Biodiversity and Ecosystem Research, Bulgarian Academy of Sciences, Sofia, Bulgaria, under the accession numbers IBER-BAS M0160.1.1-M0160.1.20; IBER-BAS M0160.2.1-M0160.2.19; and IBER-BAS M0160.3.1–3.4.

## Declaration of competing interests

The authors declare that they have no known competing financial interests or personal relationships that could have appeared to influence the work reported in this paper.

Given their role as Editor-in-Chief, Aneta Kostadinova had no involvement in the peer-review of this article and has no access to information regarding its peer-review. Full responsibility for the editorial process for this article was delegated to Co-Editor Hesham M. Al-Mekhlafi.
