# Supplementary material for: Novel molecular data for monogenean parasites of sparid fishes in the Mediterranean and a molecular phylogeny of the Microcotylidae Taschenberg, 1879
Source: Curr Res Parasitol Vector Borne Dis. 2021 Dec 24;2:100069. doi: 10.1016/j.crpvbd.2021.100069 (PMC9795350; doi:10.1016/j.crpvbd.2021.100069)
Supplement: Supplementary file 1 [file mmc1.pdf]

Supplementary Table S1. List of monogenean taxa included in the phylogenetic analyses with provenance data on their hosts, localities and GenBank accession numbers

Monogenean species	Host species	Isolate	Locality	GenBank ID *cox*1	28S rDNA	Reference
**Family Microcotylidae Taschenberg, 1879**						
**Subfamily Anchoromicrocotylinae Bravo-Hollis, 1981**						
Cynoscionicola branchialis[a]	*Umbrina xanti* Gill		Mexico		AF382050	Olson & Littlewood (2002)
**Subfamily Atriasterinae Maillard & Noisy, 1979**						
Atrispinum acarne Maillard & Noisy, 1979	*Pagellus acarne* (Risso)		Off Sète, France (Western		AF311702	Jovelin & Justine (2001)
Bychowskicotyla mormyri (Lorenz, 1878) Unnithan, 1971	*Lithognathus mormyrus* (L.)		Mediterranean)	AY009160	AF311713	
Sparicotyle chrysophrii (van Beneden & Hesse, 1863) Mamaev, 1984	*Sparus aurata* L.		Off Sète, France (Western Mediterranean)	AY009161	AF311719	Jovelin & Justine (2001)
		L4	Adriatic Sea	GQ240236		Mladineo et al. (2009)
**Subfamily Metamicrocotylinae Yamaguti, 1963**						
Intracotyle sp. SC-2018	na		na		MH700262	Chou (unpublished)
Metamicrocotyla sp. SC-2018	na		na		MH700260	
**Subfamily Microcotylinae Taschenberg, 1879**						
Bivagina pagrosomi (Murray, 1931) Dillon & Hargis, 1965	*Sparus aurata* L.		Coffs Harbor, NSW, Australia	Z83003	Z83002	Littlewood et al. (1997)
	na		na		AJ243678	Littlewood et al. (1999)
Caballeraxine sp. SC-2018					MH700261	Chou (unpublished)
Diplostamenides sciaenae (Goto, 1894) Mamaev, 1986	*Johnius belangerii* (Cuvier)		Versova Dock Landing Centre, Mumbai, India		KU204208	Verma & Agrawal (unpublished)
	na		na		FJ432589	Su (unpublished)
Diplostamenides sp. SC-2018	na		na		MH700263	Chou (unpublished)
Kahawaia truttae (Dillon & Hargis, 1965) Lebedev, 1969	*Arripis trutta* (Forster)		Off Bermagui, Australia		GU263832	Catalano et al. (2010)
	Arripis truttacea (Cuvier)		Great Australian Bight		GU263831	
Lutianicola sp. SC-2018	na		na		MH700259	Chou (unpublished)
Microcotyle algeriensis Ayadi, Gey, Justine & Tazerouti, 2016	*Scorpaena notata* Rafinesque	MO-01	Off Bouharoun, Algeria	KX926443		Ayadi et al. (2017)
		MO-02	(Western Mediterranean)	KX926444		
		MO-03		KX926445		
Microcotyle archosargi MacCallum, 1913	*Archosargus rhomboidalis* (L.)	81	Off Seybaplaya Coast, Campeche Bank, Mexico		MG586867	Mendoza-Franco et al. (2018)
Microcotyle arripis Sandars, 1945	*Arripis georgiana* (Valenciennes)		Off Australia		GU263830	Catalano et al. (2010)
Microcotyle caudata Goto, 1894	*Sebastes innermis* Cuvier	MC06	Seto Inland Sea, Japan	LC472527		Ono et al. (2020)
	“*Sebastes inermis* species	MC12		LC472528		
	complex”	MC18		LC472529		
		MC20		LC472530		
		MC24		LC472531		
Microcotyle erythrini van Beneden & Hesse, 1863	*Pagellus erythrinus* (L.)		Off France (Western		AM157221	Badets et al., (2011)
			Mediterranean)	AY009159		Jovelin & Justine (2001)
		MePe1	Off Guardamar del		MN814848	Víllora-Montero et al. (2020)
		MePe2	Segura, Spain (Western	MN816012		
			Mediterranean)	MN816013		

	Pagrus pagrus (L.)	MePp1<br>MePp2<br>MePp3<br>MePp4		MN816014<br>MN816015<br>MN816016<br>MN816017	MN814849	
Microcotyle isyebi Bougerche, Gey, Justine & Tazerouti, 2019	*Boops boops* (L.)	MO01	Off Algeria (Western Mediterranean)	MK317922		Bouguerche et al. (2019a)
		MiBb1<br>MiBb2<br>MiBb3<br>MiBb4	Off Guardamar del Segura, Spain (Western Mediterranean)	MN816018<br>MN816019<br>MN816020<br>MN816021	MN814850	Víllora-Montero et al. (2020)
Microcotyle kasago Ono, Matsumoto, Nitta & Kamio, 2020	*Sebastiscus marmoratus* (Cuvier)	MK02<br>MK01	Seto Inland Sea, Japan	LC472525<br>LC472526		Ono et al. (2020)
Microcotyle sebastis Goto, 1894	*Sebastes* sp.		North Sea, off UK		AF382051	Olson & Littlewood (2002)
	Sebastes schlegelii Hilgendorf		Fish farm, South Korea	DQ412044		Park et al. (2007)
Microcotyle visa Bougerche, Gey, Justine & Tazerouti, 2019	*Pagrus caeruleostictus* (Valenciennes)	PacoerMO01	Off Algeria (Western Mediterranean)	MK275652		Bouguerche et al. (2019b)
		PacoerMO02		MK275653		
		PacoerMO03		MK275654		
Microcotyle whittingtoni Víllora-Montero, Pérez-del-Olmo, Georgieva, Raga & Montero, 2020	*Dentex dentex* (L.)	MwDd1<br>MwDd2	Off Guardamar del Segura, Spain (Western Mediterranean)	MN816010<br>MN816011	MN814847	Víllora-Montero et al. (2020)
Microcotyle sp. 1 SC-2018	na		na		MH700256	Chou (unpublished)
Microcotyle sp. 2 SC-2018	na	SC-2018	na		MH700266	
Microcotyle sp. DG-2016	*Helicolenus dactylopterus* (Delaroche)	MO-06<br>MO-04	Off Bouharoun, Algeria (Western Mediterranean)	KX926447<br>KX926446		Ayadi et al. (2017)
Microcotyle sp.[b]	*Dentex dentex* (L.)	JWJ-2015	Off Crete, Greece		KT191025	Jun (2015)
Microcotylidae gen. sp. M10	*Sebastes* sp.		Off UK		EF653385	Aiken et al. (2007)
Microcotyloides incisus (Linton, 1910) Fujii, 1944	*Rhomboplites aurorubens* (Cuvier)	DNA-2546	Northern Gulf of Mexico, off USA		KU527427[f]	Claxton et al. (2017)
	Lutjanus griseus (L.)	210; 211<br>210	Campeche Bank, Mexico		MG586862<br>MG586861	Mendoza-Franco et al. (2018)
Omanicotyle heterospina (Mamaev & Parukhin, 1974) Yoon, Al-Jufaili, Freeman, Bron, Paladini & Shinn, 2013	*Argyrops spinifer* (Forsskål)		Off Muscat, Oman Sea		JN602095	Yoon et al. (2013)
"*Paramicrocotyle*" sp. FAS-2014	*Pinguipes chilensis* Valenciennes		Off Coquimbo, Chile	KJ794215		Oliva et al. (2014)
Paracaesicola nanshaensis Zhou, Li, Liu, Ding & Yuan, 2020[c]	*Paracaesio sordidus* Abe & Shinohara		Off China		MH700264	Zhou et al. (2020)
Pauciconfibula draconis (Briot, 1904) DilIon & Hargis, 1965	*Trachinus draco* L.	MNHN HEL1421	Off Algeria	MW484929		Azizi et al. (2021)
Pauciconfibula trachini (Parona & Perugia, 1889) Dilllon & Hargis, 1965	*Trachinus radiatus* Cuvier	TrraRamla1Pau1	Off Tunisia	MW484936		Azizi et al. (2021)
Polynemicola sp. SC-2018	na		na		MH700265	Chou (unpublished)
Polylabris sillaginae (Woolcock, 1936)	*Sillaginodes punctatus* (Cuvier)		Off Australia		GU289509	Catalano et al. (2010)
**Subfamily Prostatomicrocotylinae Yamaguti, 1968**						
Polylabris halichoeres Wang & Zhang, 1998	na		na	JF505509		Zhang et al. (2011)

Polylabroides guangdongensis Zhang & Yang, 2000	na		na	JQ038230		Zhang et al. (unpublished)
Polylabris cf. *mamaevi* SC-2018	na		na		MH700591	Chou (unpublished)
Polylabris sp. SC-2018	na		na		MH700257	
Polylabroides sp. SC-2018	na		na		MH700258	
**Family Capsalidae Baird, 1853**						
Encotyllabe antofagastensis Sepúlveda, González & Oliva, 2014	*Anisotremus scapularis* (Tschudi)	As1F74	Off Antofagasta, Chile	JQ782836		Sepúlveda et al. (2014)
		As3F135		JQ782837		
		As4F136		JQ782838		
		As2F113		JQ782839		
		As3F134		JQ782840		
		F1	Off Cabo Frio, RJ, Brazil		MT982166	Taborda et al. (unpublished)
Encotyllabe caballeroi Velasquez, 1977	*Gymnocranius audleyi* Ogilby		Off Heron Island, QLD, Australia		AF026112	Mollaret et al. (1997)
Encotyllabe caranxi Lebedev, 1967	*Pseudocaranx dentex* (Bloch & Schneider)	AHC 29665	Off Australia		FJ971990	Perkins et al. (2009)
Encotyllabe cheilodactyli Sepúlveda, González & Oliva, 2014	*Cheilodactylus variegatus* Valenciennes	Cv3F60	Off Antofagasta, Chile	JQ782841		Sepúlveda et al. (2014)
		Cv1F29		JQ782842		
		Cv1F30		JQ782843		
		Cv2F31		JQ782844		
		Cv3F61		JQ782845		
		F29	Off Cabo Frio, RJ, Brazil		MT982167	Taborda et al. (unpublished)
Encotyllabe chironemi Robinson, 1961	*Chironemus marmoratus* Günther		Coffs Harbour, Australia		AF382054	Olson & Littlewood (2002)
Encotyllabe cf. *spari* ACAC-2017	*Orthopristis ruber* (Cuvier)	E451	Off Urca, Brazil		KY553149	Camargo et al. (2017)
Encotyllabe sp. h FAS-2020		F44	Off Cabo Frio, RJ, Brazil		MT968927	Taborda et al. (unpublished)
		E1.6-E1.9		MT967362		
Encotyllabe sp. y FAS-2020	*Pagrus pagrus* (L.)	E2.2			MT968928	

[a]As "*Cynoscionicola branquialis*" (lapsus) (syn. of *C. branchialis*) on GenBank; [b]As "Microcotylidae sp. JWJ-2015" on GenBank; [c]As "Monogenea gen. n. sp. SC-2018" on GenBank.

References

Aiken, H.M., Bott, N.J., Mladineo, I., Montero, F.E., Nowak, B.F. & Hayward, C.J. (2007) Molecular evidence for cosmopolitan distribution of platyhelminth parasites of tunas (*Thunnus* spp.) *Fish & Fisheries*, 8, 167–180.

Ayadi, Z.E.M., Gey, D., Justine, J.-L. & Tazerouti, F. (2017) A new species of *Microcotyle* (Monogenea: Microcotylidae) from *Scorpaena notata* (Teleostei: Scorpaenidae) in the Mediterranean Sea. *Parasitology International*, 66, 37–42.

Azizi, R., Bouguerche, C., Santoro, M., Gey, D., Tazerouti, F., Justine, J.-L. & Bahri, S. (2021) Redescription and molecular characterization of two species of Pauciconfibula (Monogenea, Microcotylidae) from trachinid fishes in the Mediterranean Sea. Parasitology Research, 120, 2363–2377.

Badets, M., Whittington, I., Lalubin, F., Allienne, J. F., Maspimby, J.L., Bentz, S., Du Preez, L.H., Barton, D., Hasegawa, H., Tandon, V., Imkongwapang, R., Ohler, A., Combes, C. & Verneau, O. (2011) Correlating early evolution of parasitic platyhelminths to Gondwana breakup. *Systematic Biology*, 60, 762–781.

Bouguerche, C., Gey, D., Justine, J.-L. & Tazerouti, F. (2019a) Towards the resolution of the *Microcotyle erythrini* species complex: description of *Microcotyle isyebi* n. sp. (Monogenea, Microcotylidae) from *Boops boops* (Teleostei, Sparidae) off the Algerian coast. *Parasitology Research*, 118, 1417–1428.

Bouguerche, C., Gey, D., Justine, J-L. & Tazerouti, F. (2019b) *Microcotyle visa* n. sp. (Monogenea: Microcotylidae), a gill parasite of *Pagrus caeruleostictus* (Valenciennes) (Teleostei: Sparidae) off the Algerian coast, Western Mediterranean. *Systematic Parasitology*, 96,131–147.

Camargo, A.C.A., Luque, J. L. & Santos, C.P. (2017) *Mexicana rubra* sp. nov. and *Encotyllabe* cf. *spari* Yamaguti, 1934 (Monogenea) of *Orthopristis ruber* (Cuvier, 1830) from the Brazilian Coast off Rio de Janeiro. *Helminthologia*, 54, 336–347.

Catalano, S.R., Hutson, K.S., Ratcliff, R.M. & Whittington, I.D. (2010) Redescriptions of two species of microcotylid monogeneans from three arripid hosts in southern Australian waters. *Systematic Parasitology*, 76, 211–222.

Claxton, A.T., Fuehring, A.D., Andres, M.J., Moncrief, T.D. & Curran, S.S. (2017) Parasites of the vermilion snapper, *Rhomboplites aurorubens* (Cuvier), from the Western Atlantic Ocean. *Comparative Parasitology*, 84, 1–14.

Jovelin, R. & Justine, J.-L. (2001) Phylogenetic relationships within the polyopisthocotylean monogeneans (Platyhelminthes) inferred from partial 28S rDNA sequences *International Journal for Parasitology*, 31, 393–401.

Jun, J.W. (2015) Phylogenetic study on *Microcotyle* sp. (Monogenea) from common dentex (*Dentex dentex*) in the Mediterranean Sea, Greece. *African Journal of Biotechnology*, 14, 2532–2538

Littlewood, D.T.J., Rohde, K. & Clough, K.A. (1997) Parasite speciation within or between host species? – phylogenetic evidence from site-specific polystome monogeneans. *International Journal for Parasitology*, 27, 1289–1297.

Littlewood, D.T.J., Rohde, K., Bray, R.A. & Herniou, E.A. (1999) Phylogeny of the Platyhelminthes and the evolution of parasitism. *Biological Journal of the Linnaean Society London*, 68, 257–287.

Mendoza-Franco, E.F., Tun, M.C.R., Anchevida, A.J.D. & Rodriguez, R.E.R. (2018) Morphological and molecular (28S rRNA) data of monogeneans (Platyhelminthes) infecting the gill lamellae of marine fishes in the Campeche Bank, southwest Gulf of Mexico. *ZooKeys*, 783, 125–161.

Mladineo, I., Šegvić, T. & Grubišić, L. (2009). Molecular evidence for the lack of transmission of the monogenean *Sparicotyle chrysophrii* (Monogenea, Polyopisthocotylea) and isopod *Ceratothoa oestroides* (Crustacea, Cymothoidae) between wild bogue (*Boops boops*) and cage-reared sea bream (*Sparus aurata*) and sea bass (*Dicentrarchus labrax*). *Aquaculture*, 295, 160–167.

Mollaret, I., Jamieson, B.G., Adlard, R.D., Hugall, A., Lecointre, G., Chombard, C. & Justine, J.-L. (1997) Phylogenetic analysis of the Monogenea and their relationships with Digenea and Eucestoda inferred from 28S rDNA sequences. *Molecular & Biochemical Parasitology*, 90, 433–438.

Oliva, M.E., Sepulveda, F.A. & Gonzalez, M.T. (2014) *Parapedocotyle prolatili* gen. n. et sp. n., a representative of a new subfamily of the Diclidophoridae (Monogenea), a gill parasite of *Prolatilus jugularis* (Teleostei: Pinguipedidae) from Chile. *Folia Parasitologica*, 61, 543–548.

Olson, P.D. & Littlewood, D.T.J. (2002) Phylogenetics of the Monogenea - evidence from a medley of molecules. *International Journal for Parasitology*, 32, 233–244.

Ono, N., Matsumoto, R., Nitta, M. & Kamio, Y. (2020) Taxonomic revision of *Microcotyle caudata* Goto, 1894 parasitic on gills of sebastids (Scorpaeniformes: Sebastidae), with a description of *Microcotyle kasago* n. sp. (Monogenea: Microcotylidae) from off Japan. *Systematic Parasitology*, 97, 501–516.

Park, J.K., Kim, K.H., Kang, S., Kim, W., Eom, K.S. & Littlewood, D.T.J. (2007) A common origin of complex life cycles in parasitic flatworms: evidence from the complete mitochondrial genome of *Microcotyle sebastis* (Monogenea: Platyhelminthes). *BMC Evolutionary Biology*, 7, 11.

Perkins, E.M., Donnellan, S.C., Bertozzi, T., Chisholm, L.A. & Whittington, I.D. (2009) Looks can deceive: molecular phylogeny of a family of flatworm ectoparasites (Monogenea: Capsalidae) does not reflect current morphological classification. *Molecular Phylogenetics & Evolution*, 52, 705–714.

Sepúlveda, F.A., Gonzalez, M.T. & Oliva, M.E. (2014) Two new species of *Encotyllabe* (Monogenea: Capsalidae) based on morphometric and molecular evidence: parasites of two inshore fish species of northern Chile. *Journal of Parasitology*, 100, 344–349.

Víllora-Montero, M., Pérez-del-Olmo, A., Georgieva, S., Raga, J.A. & Montero, F.E. (2020) Considerations on the taxonomy and morphology of *Microcotyle* spp.: redescription of *M. erythrini* van Beneden & Hesse, 1863 (*sensu stricto*) (Monogenea: Microcotylidae) and the description of a new species from *Dentex dentex* (L.) (Teleostei: Sparidae). *Parasites & Vectors*, 13, 45.

Yoon, G.H., Al-Jufaili, S., Freeman, M.A., Bron, J.E., Paladini, G. & Shinn, A.P. (2013) *Omanicotyle heterospina* n. gen. et n. comb. (Monogenea: Microcotylidae) from the gills of *Argyrops spinifer* (Forsskal) (Teleostei: Sparidae) from the Sea of Oman. *Parasites & Vectors*, 6, 170.

Zhang, J., Wu, X., Xie, M., Xu, X., & Li, A. (2011) The mitochondrial genome of *Polylabris halichoeres* (Monogenea: Microcotylidae). *Mitochondrial DNA*, 22, 3–5.

Zhou, Z.-H., Li, Y.-Z., Lui, L., Ding, X.-J. & Yuan, K. (2020) *Paracaesicola nanshaensis* n. gen., n. sp. (Monogenea, Microcotylidae) a gill parasite of *Paracaesio sordida* (Teleostei, Lutjanidae) from the South China Sea. *Parasite* 27, 33.
