# Supplementary material for: Novel molecular data for monogenean parasites of sparid fishes in the Mediterranean and a molecular phylogeny of the Microcotylidae Taschenberg, 1879
Source: Curr Res Parasitol Vector Borne Dis. 2021 Dec 24;2:100069. doi: 10.1016/j.crpvbd.2021.100069 (PMC9795350; doi:10.1016/j.crpvbd.2021.100069)
Supplement: Supplementary file 2 [file mmc2.pdf]

Supplementary Table S2. Haplotypes of *Sparicotyle chrysophrii*

Host	Isolate	GenBank ID	Haplotype	Reference
Sparus aurata	L5	GQ240237	H1	Mladineo et al. (2009)
	L7	GQ240239	H1	
	L1	GQ240240	H1	
	L2	GQ240241	H1	
	L3	GQ240242	H1	
	L8	GQ240243	H1	
	L9	GQ240244	H1	
	M14	GQ240249	H1	
	L10	GQ240245	H1	
	M16	GQ240255	H1	
	M13	GQ240254	H1	
Boops boops	M18	GQ240256	H2	
	M19	GQ240257	H2	
	M20	GQ240258	H2	
	M21	GQ240259	H2	
	M22	GQ240260	H2	
	L23	GQ240261	H2	
	L24	GQ240262	H2	
	L25	GQ240263	H2	
	L26	GQ240264	H2	
	L27	GQ240265	H2	
Sparus aurata	L4	GQ240236	H3	
	L6	GQ240238	H3	
	M15	GQ240250	H3	
	M8	GQ240246	H4	
	M7	GQ240251	H4	
	M10	GQ240248	H5	
	M12	GQ240253	H5	
	M9	GQ240247	H6	
	M11	GQ240252	H6	
Sparus aurata	JJ	AY009161	H7	Jovelin & Justine (2001)
Sparus aurata	MR 82	OL675207	H8	Present study
	MR 79	OL675206	H9	
	MR 51	OL675208	H10	
	MR 57	OL675210	H11	
	MR 69	OL675209	H12	

References

Jovelin, R. & Justine, J.-L. (2001) Phylogenetic relationships within the polyopisthocotylean monogeneans (Platyhelminthes) inferred from partial 28S rDNA sequences *International Journal for Parasitology*, 31, 393–401.

Mladineo, I., Šegvić, T. & Grubišić, L. (2009) Molecular evidence for the lack of transmission of the monogenean *Sparicotyle chrysophrii* (Monogenea, Polyopisthocotylea) and isopod *Ceratothoa oestroides* (Crustacea, Cymothoidae) between wild bogue (*Boops boops*) and cage-reared sea bream (*Sparus aurata*) and sea bass (*Dicentrarchus labrax*). *Aquaculture*, 295, 160–167.
